# Paddington Green Children's Hospital

**Published:** 1893-06-10

**Authors:** 


### Festival Dinners and Meetings.

### PADDINGTON GREEN CHILDREN'S HOSPITAL.

The tenth annual meeting of the Committee was held at the Hospital on Wednesday, the 31st ult. In the absence of Mr. Hanbury, the chair was taken by the Rev. C. J. Ridgeway. Letters of regret for their inability to attend were read by Dr. Leslie Ogilvie from Lord Randolph Churchill and others.

The report for 1892 showed that in consequence of overcrowding and general unsanitary conditions various alterations were found to be necessary. These accomplished, the hospital was reopened in April, but a repetition of the same troubles caused the medical staff to present a special report in June, in which it was declared that the hospital was totally unworkable under present conditions. Accordingly the in-patient department was closed, and a house near Harrow was taken for the reception of patients, 65 children being admitted before the end of the year. After serious consideration the Committee decided that the only course left to pursue was to pull down the present building and erect an entirely new one. Plans have been prepared, Messrs. Salter and Adams being appointed architects. The new building will contain all the most modern improvements, the estimated cost, including that of furnishing, being £12,000, of which about £7,000 are in hand. The Committee earnestly appeal for funds to enable them to carry out the work as it should be done. The work of the Convalescent Home at Wembley has gone on satisfactorily during the year; 27 children were admitted.

The report was adopted, and the meeting closed with the usual votes of thanks.

